# Development and multicenter validation of a predictive model for malignant pleural effusion recurrence

**DOI:** 10.1016/j.isci.2026.115040

**Published:** 2026-02-17

**Authors:** Xin Hu, Yongjie Jiang, Yiluo Heibi, Li Jiang, Yuying Li

**Affiliations:** 1 Department of Respiratory and Critical Care Medicine, Inflammations & Allergic Diseases Research Unit, The Affiliated Hospital of Southwest Medical University, Luzhou, China; 2 Department of Respiratory and Critical Care Medicine, Chengdu Third People’s Hospital, Chengdu, China; 3 Department of Respiratory and Critical Care Medicine, Guang’an People’s Hospital, Guang’an, China; 4 Department of Respiratory and Critical Care Medicine, Affiliated Hospital of North Sichuan Medical College, Nanchong, China

**Keywords:** health sciences, medicine, medical specialty, oncology, health technology

## Abstract

Early prediction of malignant pleural effusion (MPE) recurrence within 3 months is essential for optimizing management in lung cancer patients. This study developed and validated a machine learning model to estimate the 3-month recurrence risk of MPE in patients with newly diagnosed lung cancer. Using data from 221 patients for model training and 237 from two external validation cohorts, the Elastic Net model—based solely on four routine clinical features (treatment regimen, alanine aminotransferase, total pleural effusion volume, and tumor diameter)—achieved excellent performance, with areas under the curve of 0.848 and 0.940 in external validation. The model significantly outperformed other machine learning approaches. An interactive risk stratification tool was further developed to classify patients into four risk groups, enabling early identification and individualized management of high-risk patients. This tool offers a practical and generalizable solution for guiding clinical decision-making.

## Highlights

• Developed a machine learning model to predict 3-month MPE recurrence in lung cancer patients
• The Elastic Net model uses only four routine clinical features
• The model achieved excellent external validation performance with AUCs of 0.848 and 0.940
• An interactive risk stratification tool was developed for individualized management

## Graphical abstract

**Figure:**
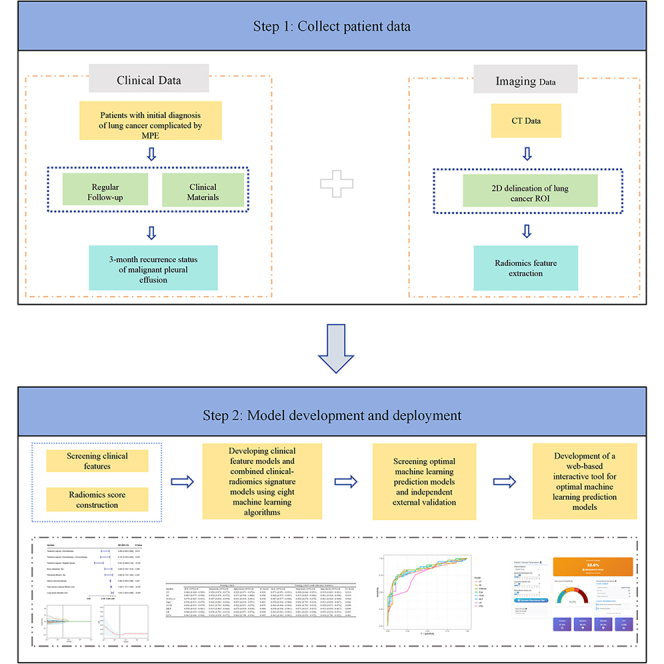

## Introduction

Malignant pleural effusion (MPE) is a common complication of advanced lung cancer, closely associated with poor patient prognosis.^1^ MPE most frequently occurs in lung cancer, especially advanced stages, followed by breast cancer, lymphoma, gynecological cancers, and malignant mesothelioma.^2^^,^^3^ The presence of MPE at the initial diagnosis of lung cancer not only significantly reduces overall survival but also complicates treatment strategies.^4^^,^^5^

Although systemic therapy and local pleural interventions can achieve initial control of MPE, recurrence after initial treatment remains a common and clinically significant outcome. MPE recurrence signifies disease progression, exacerbates symptoms, often necessitates repeated invasive procedures, and further diminishes survival expectancy and quality of life.^6^ Early identification of patients at high risk of recurrence enables intensified monitoring, prophylactic interventions, individualized treatment escalation, and optimized palliative care planning.

Current prognostic models for lung cancer primarily focus on overall survival or initial treatment response prediction. Research specifically targeting risk stratification for MPE recurrence after completion of initial treatment is remains limited.^7^^,^^8^ Moreover, high-precision models often require complex or expensive biomarker testing, limiting their practical clinical application. Feature extraction methods from medical images based on computed tomography (CT) scans have emerged as a novel approach to enhance prediction accuracy.^9^^,^^10^^,^^11^ Imaging indicators such as tumor size, texture features, and morphological changes may provide important insights into MPE recurrence risk. The application of artificial intelligence (AI), particularly deep learning, has shown great promise in revolutionizing lung cancer diagnosis. For example, state-of-the-art approaches have successfully utilized deep learning models like convolutional neural networks to analyze next-generation sequencing data, achieving high accuracy in identifying genetic biomarkers.^12^ Our study complements these genomics-focused efforts by demonstrating the significant predictive power of routinely available clinical data. Therefore, this study focused on developing a computational model using routinely accessible clinical parameters and CT radiomics features to accurately predict the probability of MPE recurrence.

## Results

### Patient features

**Figure 1:**
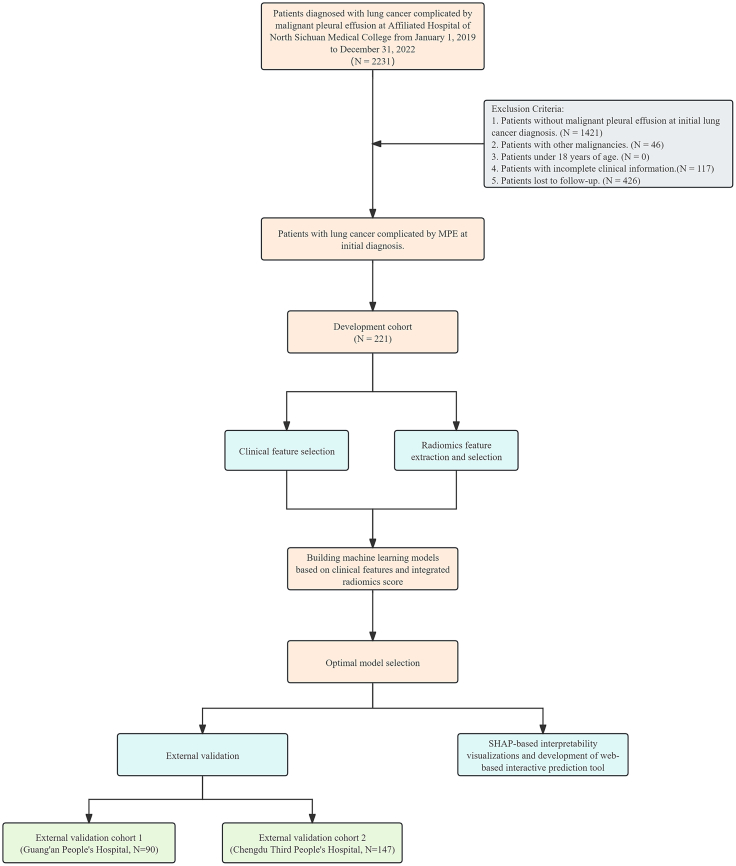
Flow chart for studying the risk factors and predictive models of recurrent MPE in patients initially diagnosed with lung cancer complicated by MPE

Based on inclusion and exclusion criteria, this study enrolled 221 patients from Affiliated Hospital of North Sichuan Medical College as the training cohort, 90 patients from Guang’an People’s Hospital as external validation cohort 1, and 147 patients from Chengdu Third People’s Hospital as external validation cohort 2 (Figure 1). In the training cohort, 103 patients (46.6%) did not experience pleural effusion recurrence beyond three months. In external validation cohort 1, 49 patients (54.4%) did not experience recurrence beyond three months. In external validation cohort 2, 77 patients (52.4%) did not experience recurrence beyond 3 months. Tables S1 and S2 detail the clinical characteristics of patients in the training and external validation cohorts.

### Clinical feature analysis and screening

**Figure 2:**
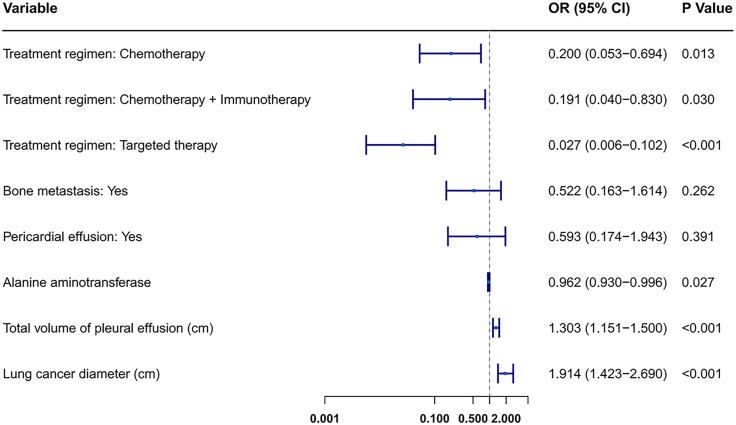
Multivariate analysis of independent predictors for 3-month MPE recurrence The forest plot displays the four independent clinical predictors retained after multivariate logistic regression analysis. For each variable, the plot shows the adjusted odds ratio (OR) with its 95% CI. An OR > 1 indicates a risk factor, while an OR < 1 indicates a protective factor.

Univariate logistic regression analysis was performed on all clinical features in the training cohort (Table 1), identifying significant features at *p* < 0.05: treatment regimen, bone metastasis, pericardial effusion, alanine aminotransferase (ALT), total volume of pleural effusion, and lung cancer diameter. Further multivariate logistic regression analysis retained features with *p* < 0.05 for model construction, ultimately identifying four core variables: treatment regimen, ALT, total volume of pleural effusion, and lung cancer diameter (Figure 2). These were incorporated into machine learning model development. Within the observed range, a higher ALT was a protective factor against MPE recurrence within 3 months, while a larger total volume of pleural effusion and larger lung cancer diameter were risk factors for MPE recurrence within 3 months.

**Table 1:** Results of univariate logistic regression analysis

	No recurrence of MPE within 3 months	Recurrence of MPE within 3 months	*p* value
n	103	118	
Gender = Woman (%)	46 (44.7)	54 (45.8)	0.977
Age = ≥60 (%)	63 (61.2)	87 (73.7)	0.064
Pathological subtype of lung cancer (%)			0.521
Adenocarcinoma	79 (76.7)	97 (82.2)	
Squamous cell carcinoma	13 (12.6)	13 (11.0)	
Small cell lung cancer	11 (10.7)	8 (6.8)	
Qualitative assessment of mucoprotein in pleural fluid = Positive (%)	99 (96.1)	112 (94.9)	0.917
Clarity of pleural effusion (%)			0.507
Clear	6 (5.8)	6 (5.1)	
Slightly turbid	40 (38.8)	55 (46.6)	
Turbid	57 (55.3)	57 (48.3)	
Pleural effusion coagulation status = Coagulation (%)	6 (5.8)	8 (6.8)	0.989
Location of pleural effusion (%)			0.372
Left side	38 (36.9)	40 (33.9)	
Right side	55 (53.4)	59 (50.0)	
Both sides	10 (9.7)	19 (16.1)	
Treatment regimen (%)			<0.001
Untreated	8 (7.8)	81 (68.6)	
Chemotherapy	18 (17.5)	23 (19.5)	
Chemotherapy + Immunotherapy	12 (11.7)	8 (6.8)	
Targeted therapy	65 (63.1)	6 (5.1)	
Smoking = Yes (%)	46 (44.7)	49 (41.5)	0.739
Drinking = Yes (%)	33 (32.0)	34 (28.8)	0.709
Family history of tumors = Yes (%)	2 (1.9)	4 (3.4)	0.806
Previous tumor history = Yes (%)	10 (9.7)	10 (8.5)	0.933
Primary tumor site = Right (%)	61 (59.2)	77 (65.3)	0.433
Cerebral infarction = Yes (%)	17 (16.5)	13 (11.0)	0.322
Hypertension = Yes (%)	15 (14.6)	23 (19.5)	0.43
Diabetes = Yes (%)	9 (8.7)	13 (11.0)	0.734
Respiratory tract infection = Yes (%)	53 (51.5)	73 (61.9)	0.155
Venous thrombosis = Yes (%)	11 (10.7)	16 (13.6)	0.655
Hypoxemia = Yes (%)	17 (16.5)	14 (11.9)	0.426
Cardiovascular disease = Yes (%)	3 (2.9)	8 (6.8)	0.313
Electrolyte imbalance = Yes (%)	36 (35.0)	41 (34.7)	1
Respiratory failure = Yes (%)	11 (10.7)	13 (11.0)	1
Acid base imbalance = Yes (%)	7 (6.8)	7 (5.9)	1
Pulmonary disease = Yes (%)	29 (28.2)	29 (24.6)	0.653
Bone metastasis = Yes (%)	38 (36.9)	24 (20.3)	0.01
Brain metastasis = Yes (%)	11 (10.7)	6 (5.1)	0.192
Adrenal metastasis = Yes (%)	2 (1.9)	3 (2.5)	1
Liver metastasis = Yes (%)	8 (7.8)	8 (6.8)	0.982
Pericardial effusion = Yes (%)	16 (15.5)	33 (28.0)	0.04
Intrapulmonary metastases = Yes (%)	29 (28.2)	43 (36.4)	0.243
High sensitivity C reactive protein in pleural fluid (mg/L), mean (SD)	13.61 (19.61)	11.01 (13.96)	0.253
Total protein in pleural fluid (g/L), mean (SD)	43.92 (8.54)	44.52 (9.82)	0.629
Adenosine deaminase in pleural fluid (U/L), mean (SD)	12.21 (9.24)	11.49 (8.37)	0.539
Glucose in pleural fluid (mmol/L), mean (SD)	6.10 (3.35)	9.74 (35.50)	0.3
Lactate dehydrogenase levels in pleural fluid (U/L), mean (SD)	656.93 (1,007.41)	611.16 (433.37)	0.654
Nucleated cell count in pleural fluid, mean (10E6/L) (SD)	2,958.17 (8,007.65)	2,239.96 (3,008.54)	0.367
Percentage of multinucleated cells in pleural fluid, mean (SD)	37.33 (91.18)	24.44 (20.52)	0.137
Percentage of mononuclear cells in pleural fluid, mean (SD)	71.46 (23.68)	74.61 (20.83)	0.294
Aspartate aminotransferase (U/L), mean (SD)	26.35 (23.52)	24.18 (13.41)	0.392
Alanine aminotransferase (U/L), mean (SD)	24.27 (29.07)	18.25 (13.23)	0.044
Albumin in pleural fluid (g/L), mean (SD)	37.04 (4.25)	36.82 (4.78)	0.721
Serum creatinine level (umol/L), mean (SD)	65.18 (23.54)	62.81 (22.65)	0.447
Neutrophil count in blood (10E9/L), mean (SD)	5.72 (2.64)	6.06 (3.80)	0.437
White blood cells (10E9/L), mean (SD)	7.74 (2.83)	8.02 (3.92)	0.557
Red blood cells (10E12/L), mean (SD)	4.27 (0.61)	4.29 (0.60)	0.766
Hemoglobin level (g/L), mean (SD)	124.28 (19.11)	126.01 (19.80)	0.512
Platelets (10E9/L), mean (SD)	245.17 (96.67)	234.71 (86.90)	0.398
Total volume of pleural effusion (cm), mean (SD)	7.69 (3.02)	13.87 (4.22)	<0.001
Lung cancer diameter (cm), mean (SD)	3.29 (1.57)	6.23 (1.85)	<0.001

### Machine learning model evaluation

Based on the 221 patients in the training cohort, chest CT image data for all these patients were obtained. Radiomics analysis extracted 1,223 radiomics features, from which 8 radiomics signatures were ultimately selected. The radiomics score was then calculated based on these 8 selected signatures (Figure S1 and Table S3). Using the clinical features and the combination of clinical features with the radscore from these 221 patients, eight machine learning models were built to predict the probability of MPE recurrence within 3 months in lung cancer patients with MPE.

**Figure 3:**
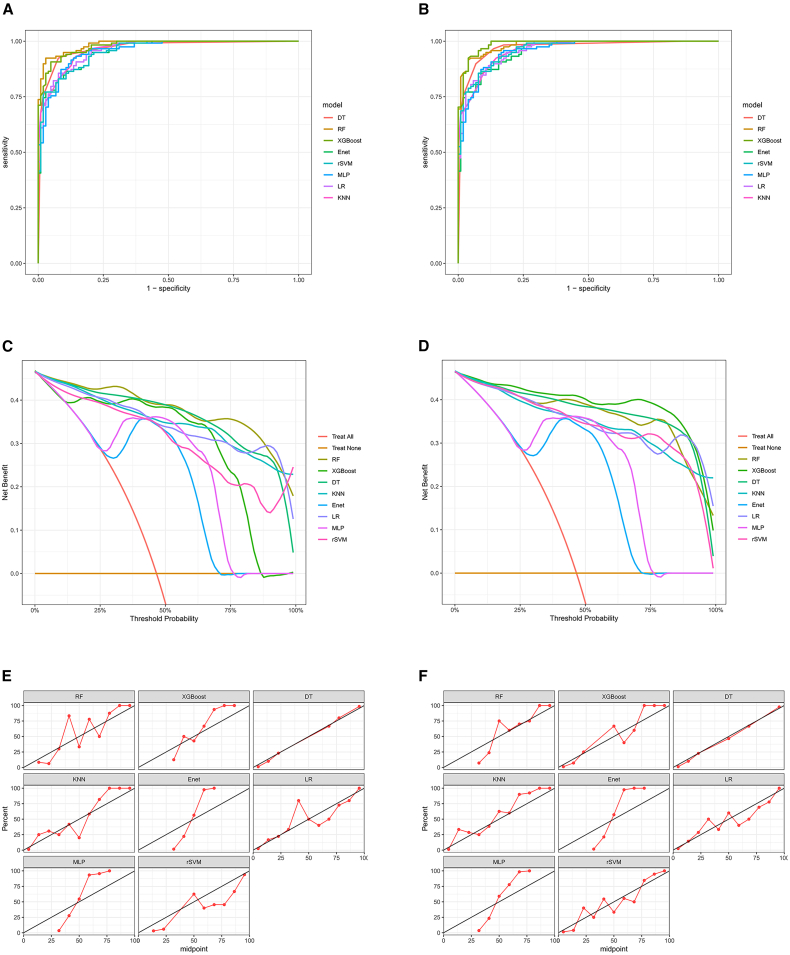
Performance comparison of prediction models in the training cohort (A) ROC curves of the eight models built on clinical features alone. (B) ROC curves of the models built on the combination of clinical features and the radiomics score. (C) DCA for the clinical feature-based models. The net benefit of using each model for clinical decision-making is plotted against the threshold probability. (D) DCA for the combined feature-based models. (E) Calibration curves for the clinical feature-based models. (F) Calibration curves for the combined feature-based models.

Results from the eight machine learning models showed: among models built on clinical features alone, the RF model had the highest area under the curve (AUC) (0.985, 95% CI: 0.975–0.996) (Figure 3A). Among models built on clinical features combined with the radiomics score, the XGBoost model had the highest AUC (0.987, 95% CI: 0.977–0.998) (Figure 3B). Notably, the difference in AUC between the eight models built on clinical features alone and those built on the combined features was not statistically significant (Table 2). Decision curve analysis (DCA) indicated that models based on both clinical features alone and the combined features provided substantial clinical benefit (Figures 3C and 3D). Calibration curves further confirmed the superior predictive performance of models built on clinical features and the combined features (Figures 3E and 3F). Furthermore, the 5-fold cross-validation performed on the training cohort confirmed the robustness of these models without signs of overfitting (Figure S2). In summary, given their simplicity and predictive accuracy, clinical features alone were chosen as the preferred indicators for building machine learning models.

**Table 2:** Performance comparison of clinical feature-based models versus clinical and radiomics feature-based models in the training cohort

Models	Training cohort					Training cohort (with radiomics features)					AUC1 vs. AUC2
	AUC1 (95% CI)	Sensitivity (95% CI)	Specificity (95% CI)	Accuracy (95% CI)	F1 Score	AUC2 (95% CI)	Sensitivity (95% CI)	Specificity (95% CI)	Accuracy (95% CI)	F1 Score	*p* value
DT	0.968 (0.948–0.989)	0.924 (0.876–0.972)	0.922 (0.871–0.974)	0.923 (0.888–0.958)	0.928	0.971 (0.951–0.991)	0.898 (0.844–0.953)	0.932 (0.883–0.981)	0.914 (0.877–0.951)	0.918	0.837
RF	0.985 (0.975–0.996)	0.924 (0.876–0.972)	0.971 (0.938–1.000)	0.946 (0.916–0.976)	0.948	0.984 (0.973–0.995)	0.915 (0.865–0.966)	0.961 (0.924–0.998)	0.915 (0.865–0.966)	0.939	0.897
XGBoost	0.979 (0.965–0.993)	0.907 (0.854–0.959)	0.951 (0.910–0.993)	0.928 (0.893–0.962)	0.930	0.987 (0.977–0.998)	0.924 (0.876–0.972)	0.961 (0.924–0.998)	0.941 (0.910–0.972)	0.944	0.370
Enet	0.956 (0.933–0.979)	0.864 (0.803–0.926)	0.893 (0.834–0.953)	0.878 (0.835–0.921)	0.883	0.958 (0.936–0.980)	0.856 (0.793–0.919)	0.913 (0.858–0.967)	0.882 (0.840–0.925)	0.886	0.902
rSVM	0.956 (0.933–0.979)	0.831 (0.763–0.898)	0.922 (0.871–0.974)	0.873 (0.829–0.917)	0.875	0.964 (0.944–0.984)	0.856 (0.793–0.919)	0.922 (0.871–0.974)	0.887 (0.845–0.929)	0.890	0.607
MLP	0.958 (0.935–0.981)	0.915 (0.865–0.966)	0.874 (0.810–0.938)	0.896 (0.856–0.936)	0.904	0.958 (0.936–0.981)	0.873 (0.813–0.933)	0.913 (0.858–0.967)	0.891 (0.850–0.932)	0.896	1.000
LR	0.960 (0.939–0.982)	0.856 (0.793–0.919)	0.922 (0.871–0.974)	0.887 (0.845–0.929)	0.890	0.961 (0.941–0.983)	0.881 (0.823–0.940)	0.893 (0.834–0.953)	0.887 (0.845–0.929)	0.893	0.948
KNN	0.965 (0.946–0.984)	0.924 (0.876–0.972)	0.864 (0.798–0.930)	0.896 (0.856–0.936)	0.905	0.963 (0.943–0.983)	0.924 (0.876–0.972)	0.854 (0.786–0.922)	0.891 (0.850–0.932)	0.901	0.887

AUC, area under the curve; DT, decision tree; RF, random forest; XGBoost, extreme gradient boosting; Enet, elastic net; rSVM, radial-basis support vector machine; MLP, multilayer perceptron; LR, logistic regression; KNN, k-nearest neighbors.

### Performance of the eight machine learning models built on clinical features in external validation

**Figure 4:**
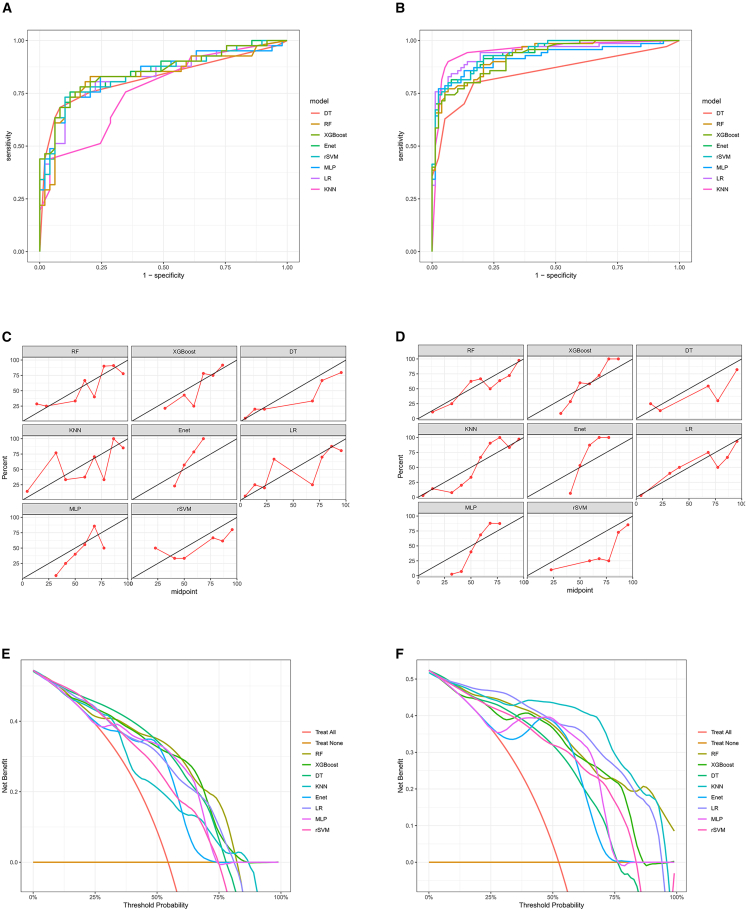
External validation of the eight clinical feature-based machine learning models (A) ROC curves demonstrating the discriminatory performance of each model in external validation cohort 1. (B) ROC curves for the models in external validation cohort 2. (C) Calibration curves assessing the prediction accuracy of the models in external validation cohort 1. (D) Calibration curves for the models in external validation cohort 2. (E) DCA evaluating the clinical utility of the models in external validation cohort 1. The net benefit of model-guided intervention is plotted against various threshold probabilities. (F) DCA for the models in external validation cohort 2.

In external validation cohort 1, elastic net (Enet) (AUC = 0.848 [95% CI: 0.764–0.931]) and eXtreme Gradient Boosting (XGBoost) (AUC = 0.851 [95% CI: 0.768–0.935]) demonstrated leading discriminatory ability (Figure 4A and Table 3). In external validation cohort 2, although K-Nearest Neighbors (KNN) had the highest AUC (0.952, 95% CI: 0.916–0.989), its cross-center stability was slightly lower (Figure 4B and Table 3). Calibration curves also showed high concordance between the predicted probabilities of all eight models and the actual observed MPE recurrence probability (Figures 4C and 4D). Furthermore, DCA performed on external validation cohorts 1 and 2 demonstrated that using any of the eight machine learning prediction models to assess the risk of MPE recurrence within 3 months and intervene accordingly provided greater clinical benefit than intervening in all patients or no intervention, within a reasonable threshold probability range (Figures 4E and 4F). Considering that Enet maintained high performance across both independent cohorts with stable specificity and the strongest generalization ability, the Enet model was established as the optimal prediction model.

**Table 3:** Performance of eight clinical feature-based machine learning predictive models in two external validation cohorts

Models	External validation cohort 1					External validation cohort 2				
	AUC (95% CI)	Sensitivity (95% CI)	Specificity (95% CI)	Accuracy (95% CI)	F1 score	AUC (95% CI)	Sensitivity (95% CI)	Specificity (95% CI)	Accuracy (95% CI)	F1 score
DT	0.822 (0.734–0.910)	0.625 (0.475–0.775)	0.939 (0.872–1.000)	0.798 (0.714–0.881)	0.735	0.847 (0.783–0.910)	0.629 (0.515–0.742)	0.948 (0.898–0.998)	0.796 (0.731–0.861)	0.746
RF	0.831 (0.739–0.923)	0.537 (0.384–0.689)	0.939 (0.872–1.000)	0.756 (0.667–0.844)	0.667	0.929 (0.891–0.968)	0.714 (0.608–0.820)	0.961 (0.918–1.000)	0.844 (0.785–0.902)	0.813
XGBoost	0.851 (0.768–0.935)	0.537 (0.384–0.689)	0.949 (0.893–1.000)	0.780 (0.699–0.861)	0.667	0.920 (0.880–0.961)	0.700 (0.593–0.807)	0.974 (0.938–1.000)	0.844 (0.785–0.902)	0.810
Enet	0.848 (0.764–0.931)	0.585 (0.435–0.736)	0.939 (0.872–1.000)	0.778 (0.692–0.864)	0.706	0.940 (0.906–0.975)	0.729 (0.624–0.833)	0.974 (0.938–1.000)	0.857 (0.801–0.914)	0.829
rSVM	0.842 (0.756–0.928)	0.488 (0.335–0.641)	0.939 (0.872–1.000)	0.733 (0.642–0.825)	0.625	0.941 (0.907–0.975)	0.671 (0.561–0.781)	0.987 (0.962–1.000)	0.837 (0.777–0.896)	0.797
MLP	0.841 (0.754–0.928)	0.610 (0.460–0.759)	0.918 (0.842–0.995)	0.778 (0.692–0.864)	0.714	0.917 (0.869–0.966)	0.743 (0.640–0.845)	0.974 (0.938–1.000)	0.864 (0.809–0.919)	0.839
LR	0.831 (0.743–0.920)	0.537 (0.384–0.689)	0.898 (0.813–0.983)	0.733 (0.642–0.825)	0.647	0.941 (0.900–0.981)	0.757 (0.657–0.858)	0.974 (0.938–1.000)	0.871 (0.817–0.925)	0.848
KNN	0.763 (0.665–0.861)	0.634 (0.487–0.782)	0.714 (0.588–0.841)	0.678 (0.581–0.774)	0.642	0.952 (0.916–0.989)	0.900 (0.830–0.970)	0.935 (0.880–0.990)	0.918 (0.874–0.963)	0.913

AUC, area under the curve; DT, decision tree; RF, random forest; XGBoost, extreme gradient boosting; Enet, elastic net; rSVM, radial-basis support vector machine; MLP, multilayer perceptron; LR, logistic regression; KNN, *k*-nearest neighbors.

### SHapley additive exPlanations (SHAP) interpretability and development of interactive tool

**Figure 5:**
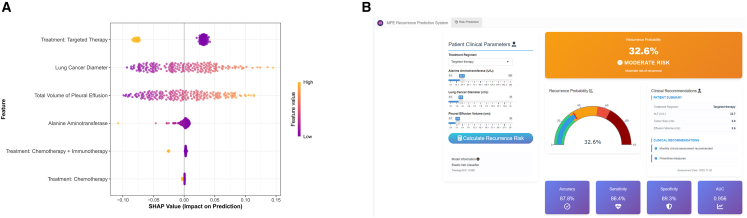
Model interpretability and the developed interactive clinical tool (A) SHAP beeswarm plot for the optimal Enet model, illustrating the impact of the four key features on the model’s output. (B) Screenshot of the interactive web-based risk prediction tool. Clinicians can input the four predictor values to receive a real-time calculation of the individualized 3-month recurrence probability, which is automatically categorized into a risk stratum with corresponding clinical management recommendations.

This study developed an SHAP beeswarm plot for the prediction model (Figure 5A), showing that targeted therapy was the strongest protective factor reducing pleural effusion recurrence risk, while larger lung cancer diameter was the primary risk factor. Additionally, after risk grading patients in the training set using the Enet model, three critical thresholds were established: (1) the recurrence probability corresponding to the model’s maximum sensitivity; (2) the recurrence probability corresponding to the maximum sum of sensitivity and specificity; and (3) the recurrence probability corresponding to the model’s maximum specificity. Patients were thus divided into four risk groups: low-risk group (recurrence probability ≤ 29%), medium-risk group (recurrence probability 29%–54%), high-risk group (recurrence probability 54%–65%), and very-high-risk group (recurrence probability >65%). To enhance clinical operability, an interactive prediction tool based on Shiny was developed based on this stratification (Figure 5B) (https://ericqazxsw.shinyapps.io/123456789/). Clinicians can input patient features to obtain individualized risk probability and graded management recommendations in real-time: Very-high-risk and high-risk groups require weekly monitoring and consideration of adjuvant intrapleural therapy; the medium-risk group is recommended for monthly clinical assessment and preventive measures; the low-risk group follows routine 3-month follow-up and is instructed on self-monitoring.

## Discussion

In lung cancer patients with MPE, recurrence signifies disease progression and is closely associated with overall survival. This study developed and validated the first stratification tool specifically designed to predict the risk of MPE recurrence within 3 months in lung cancer patients newly diagnosed with MPE. By integrating routine clinical parameters—including treatment regimen, primary tumor diameter, total pleural effusion volume, and ALT level—a pure clinical feature model based on the Enet algorithm demonstrated excellent performance in external multicenter validation. Notably, the incorporation of radiomics features did not significantly enhance predictive accuracy compared to the model using clinical features alone. Interpretability analysis using SHAP revealed that targeted therapy was the strongest protective factor against MPE recurrence, while larger tumor diameter was the primary risk factor.

Previous studies have identified several biomarkers associated with MPE recurrence, such as elevated neutrophil-to-lymphocyte ratio, serum lactate dehydrogenase, and serum carcinoembryonic antigen levels, while also noting the potential protective role of targeted therapy.^11^ Radiomics features have also been explored for predicting MPE recurrence.^12^ Although numerous high-precision predictive models exist for MPE prognosis, most focus on overall survival or initial treatment response.^13^^,^^14^^,^^15^ Research specifically addressing the prediction of 3-month recurrence risk in patients newly diagnosed with MPE remains scarce. Our study fills this gap by leveraging routinely available clinical data to build a highly generalizable and interpretable model.

This study confirmed that treatment regimen, lung cancer diameter, total pleural effusion volume, and ALT level are key independent predictors of MPE recurrence. Targeted therapy precisely inhibits driver genes, blocking tumor cell secretion of pro-angiogenic factors such as vascular endothelial growth factor/interleukin-6, thereby significantly reducing pleural microvascular leakage.^16^^,^^17^ A larger primary tumor diameter not only mechanically compresses mediastinal lymphatics, impairing fluid drainage, but is also associated with heightened epithelial-mesenchymal transition activity, which promotes pleural implantation of tumor cells.^18^^,^^19^^,^^20^ Increased total pleural effusion volume creates a vicious cycle by compressing lung tissue and reducing lymphatic drainage efficiency. Moreover, the rich transforming growth factor β content in the effusion induces cancer-associated fibroblasts to secrete hyaluronic acid, increasing colloid osmotic pressure and promoting fibrous encapsulation.^21^ Interestingly, moderately elevated ALT was identified as a protective factor against MPE recurrence. We speculate that this may reflect an active hepatic metabolic state, potentially enhancing the clearance of inflammatory mediators and optimizing the metabolism of targeted drugs, thereby indirectly inhibiting MPE recurrence in the absence of severe liver pathology.

In addition to these core factors, univariate analysis revealed that the presence of bone metastasis and pericardial effusion were also significantly associated with MPE recurrence. Bone metastasis often signifies a higher systemic tumor burden and more aggressive disease biology, which could predispose patients to pleural dissemination and fluid reaccumulation. Similarly, pericardial effusion may reflect extensive serosal involvement or compromised thoracic lymphatic drainage, creating a milieu conducive to pleural fluid recurrence. Although these variables did not retain independent significance in the multivariate model—likely due to collinearity with or being overshadowed by the stronger predictors like tumor diameter and treatment regimen—their univariate significance underscores the close interplay between multi-cavity effusions and advanced tumor progression in driving recurrence risk. Notably, several demographic and baseline clinical characteristics, such as gender, pathological subtype, and most comorbidities, showed no significant association. This suggests that the risk of early MPE recurrence is driven primarily by tumor burden and treatment-related factors rather than by patient demographics or common comorbid conditions. Moreover, age ≥60 years showed a borderline association with recurrence risk. Elderly patients may exhibit reduced immune surveillance and lower tolerance to systemic therapy, which could predispose them to earlier recurrence—an observation that warrants further validation in larger cohorts.

Our findings on predictive clinical features for MPE recurrence align with the broader trend of AI-driven biomarker discovery. As highlighted in recent literature, AI serves as a powerful tool for deciphering complex patterns in biomedical data to identify novel biomarkers and enhance diagnostic frameworks.^22^ The success of our model, which relies solely on easily accessible clinical parameters, underscores the translational potential of AI in creating practical tools for personalized patient management.

To sum up, the Enet model based on routine clinical indicators demonstrated robust performance in predicting the 3-month recurrence risk of MPE in lung cancer patients. To bridge the gap between model development and clinical practice, we developed an interactive Shiny-based risk stratification tool. This tool facilitates real-time risk assessment and supports individualized management, thereby significantly lowering the threshold for clinical deployment. It offers a practical and universally accessible solution, particularly for resource-limited institutions, with the potential to guide early intervention in high-risk patients.

### Limitations of the study

This study has several limitations. First, the retrospective design may introduce confounding bias and preclude causal inferences, necessitating future prospective validation. Second, although the model was externally validated across multiple hospitals, all centers were within the same geographic region of China, which may limit its generalizability to other populations. Therefore, international cohort validation is therefore warranted. Third, the 2D region of interest (ROI) delineation method, while simple and practical, cannot fully capture the three-dimensional morphological heterogeneity of the tumor. Finally, the model is currently limited to clinical and radiomic features. As the field of AI in lung cancer is increasingly moving toward multiomics frameworks, future work should integrate molecular data from sources such as next-generation sequencing to capture the biological drivers of MPE recurrence and enhance the model’s predictive power and interpretability.^23^

## Resource availability

### Lead contact

Requests for further information and resources should be directed to and will be fulfilled by the lead contact, Yuying Li (lzhlyyhy@126.com).

### Materials availability

This study did not generate new unique materials.

### Data and code availability

• De-identified clinical data reported in this paper have been deposited at Zenodo and are publicly available as of the date of publication at https://doi.org/10.5281/zenodo.18391732.
• This paper does not report original code.
• Any additional information required to reanalyze the data reported in this paper is available from the lead contact upon request.

## Acknowledgments

This research was supported by the Foundation of Tianfu Emei Plan (no. CW202204), the 10.13039/501100018542Natural Science Foundation of Sichuan Province (no. 2023NSFSC0527), and Science and Technology Cooperation Project between Luzhou Municipal Government and 10.13039/501100014895Southwest Medical University (2024LZXNYDJ003).

## Author contributions

Conceptualization, X.H., Y.J., L.J., and Y.L.; methodology, X.H. and Y.J.; investigation, X.H., Y.J., Y.H., and Y.L.; writing – original draft, X.H. and Y.J.; writing – review and editing, all authors; supervision, Y.H., L.J., and Y.L.

## Declaration of interests

All authors declare no competing interests.

## Declaration of generative AI and AI-assisted technologies in the writing process

Not applicable.

## STAR★Methods

### Key resources table

**Table:** 

REAGENT or RESOURCE	SOURCE	IDENTIFIER
De-identified clinical data	Zenodo	https://doi.org/10.5281/zenodo.18391732
R (version 4.4.1)	The R Foundation	https://cran.r-project.org/bin/windows/base/old/4.4.1/
3D Slicer (version 5.8.1)	Slicer Community	https://www.slicer.org/
Python (version 3.11.5)	Python Software Foundation	https://www.python.org/

### Experimental model and study participant details

#### Study population

This multicenter, retrospective study enrolled a total of 458 human participants diagnosed with lung cancer and presented with malignant pleural effusion (MPE) at initial diagnosis. Data were collected from patients treated between January 1, 2019, and December 31, 2022, at three medical centers in China: the Affiliated Hospital of North Sichuan Medical College (training cohort, n=221), Guang'an People's Hospital (external validation cohort 1, n=90), and Chengdu Third People's Hospital (external validation cohort 2, n=147). Detailed demographic data were available for the training cohort, which consisted of 121 males (54.8%) and 100 females (45.2%), with 150 patients (67.9%) aged ≥60 years. All participants were of Chinese ethnicity. For the two external validation cohorts, data collection was focused on the specific clinical and radiomic variables required for model validation; thus, full demographic breakdowns (sex and age) were not recorded for these subsets.

#### Inclusion and exclusion criteria

Inclusion Criteria: (1) Patients with a histopathologically confirmed diagnosis of lung cancer, including non-small cell lung cancer and small cell lung cancer; (2) Diagnosis of MPE confirmed either by cytological/histopathological assessment of the effusion or pleural tissue, OR by meeting the following clinical-imaging criteria: (a) histopathologically confirmed lung cancer with no other reasonable cause for the effusion, or (b) confirmed lung cancer with pleural effusion and imaging evidence (CT, positron emission tomography - computed tomography, magnetic resonance imaging or ultrasound) of pleural nodules, masses, or irregular thickening consistent with pleural metastasis. Exclusion Criteria: (1) Lung cancer patients without MPE at initial diagnosis; (2) Individuals with a history of other malignancies; (3) Patients under 18 years of age; (4) Cases with incomplete clinical information; (5) Patients lost to follow-up.

#### Ethical statement

The study protocol was approved by the Medical Ethics Committee of the Affiliated Hospital of North Sichuan Medical College (Approval File Number: 2023ER423-1). The study was conducted in accordance with the ethical principles of the Declaration of Helsinki. Due to the retrospective nature of the study, which utilized anonymized data from hospital electronic medical records and health check databases, the requirement for informed consent was waived by the approving ethics committee.

### Method details

#### Screening of clinical features

In the training cohort, all variables were first analyzed using univariate logistic regression. Variables with a *p*-value < 0.05 were then included in multivariate logistic regression analysis to identify independent predictors. Variables retaining a *p*-value < 0.05 in the multivariate analysis were retained for subsequent model development.

#### Image evaluation and feature extraction

Delineation of the ROI for the lung cancer lesion was performed by two physicians using 3D Slicer software on the training cohort scans. Discrepancies in delineation were resolved through consultation with a third physician. Radiomics features were then extracted from the delineated ROIs again using 3D Slicer, yielding a total of 1223 features. In the preliminary analysis, Mann-Whitney U tests were performed on the imaging features using Python (version 3.11.5). Features with a significance level of *p* < 0.05 were standardized using *Z*-Score normalization and further screened using the least absolute shrinkage and selection operator (LASSO) algorithm, ultimately identifying 8 radiomics signatures. The radiomics score was calculated based on these 8 signatures.

#### Machine learning model construction

Based on the aforementioned clinical features and radiomics features, two types of prediction models were constructed: pure clinical feature models and clinical feature combined with radiomics score models. The selected features were input into eight machine learning algorithms for modeling: Decision Tree (DT), Random Forest (RF), XGBoost, Radial-basis Support Vector Machine (rSVM), Multilayer Perceptron (MLP), Logistic Regression (LR), KNN, and Enet. Specific hyperparameter tuning strategies and parameter ranges for these algorithms are detailed in Methods S1. All models were trained using 5-fold cross-validation and their performance was evaluated on both the training set and the external validation sets.

### Quantification and statistical analysis

Model performance was evaluated using the receiver operating characteristic curve (ROC), calculating sensitivity, specificity, accuracy, F1 score, and AUC. Calibration curves assessed the agreement between predicted probabilities and observed probabilities, reflecting model calibration. DCA quantified the net benefit of the model compared to alternative diagnostic/prognostic strategies, evaluating clinical utility. Finally, an interactive risk prediction system for the optimal model was built using Shiny. Except for the methods mentioned above, all analyses were performed in the R (version 4.4.1) environment.

## Supplemental information

Document S1. Figures S1 and S2, Tables S2 and S3, and Methods S1

Table S1. Baseline features of the training cohort

Data S1. Ethical approval documents
