# Supplementary material for: Development and multicenter validation of a predictive model for malignant pleural effusion recurrence
Source: iScience. 2026 Feb 17;29(3):115040. doi: 10.1016/j.isci.2026.115040 (PMC12989844; doi:10.1016/j.isci.2026.115040)
Supplement: Document S1. Figures S1 and S2, Tables S2 and S3, and Methods S1 [file mmc1.pdf]

iScience, Volume *29*

Supplemental information

Development and multicenter validation of a predictive model for malignant pleural effusion recurrence

Xin Hu, Yongjie Jiang, Yiluo Heibi, Li Jiang, and Yuying Li

SUPPLEMENTARY FIGURES

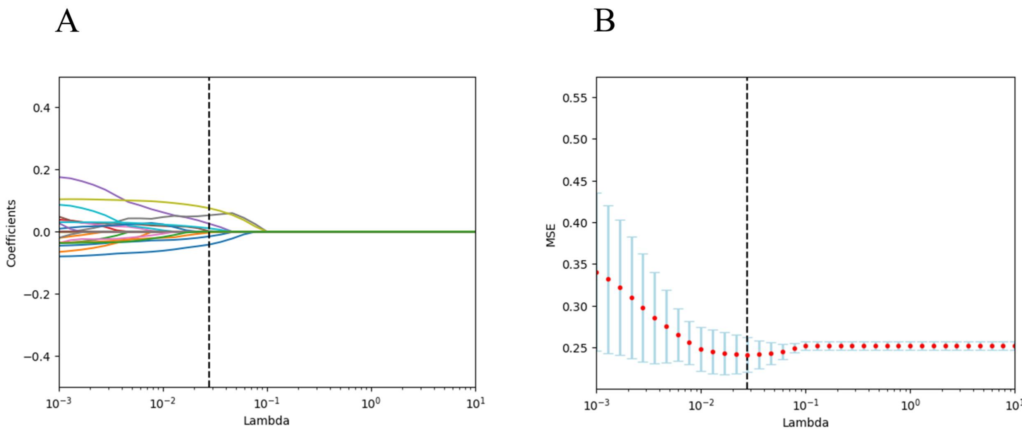

Figure S1. Identification of essential radiomic features within the lung cancer region using LASSO regression analysis.

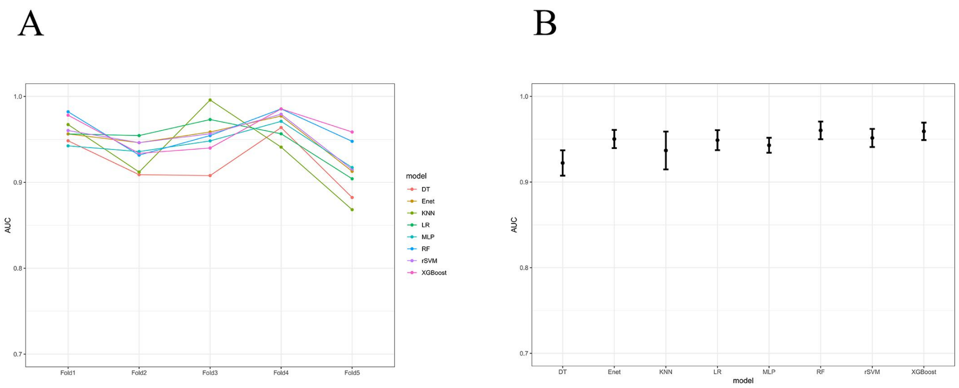

Figure S2.Cross-validation performance of the eight models in the training cohort. (A) AUC fluctuation across the five folds; (B) Distribution of mean AUC values for the models (error bars indicate standard error).

SUPPLEMENTARY TABLES

Table S2. Baseline features of the two external validation cohorts.

Features	Level	External validation cohort 1 (%) (*n*=90)	External validation cohort 2 (%) (*n*=147)
Status (%)	No recurrence of MPE within 3 months	49 (54.4)	77 (52.4)
	Recurrence of MPE within 3 months	41 (45.6)	70 (47.6)
Treatment regimen (%)	Untreated	26 (28.9)	58 (39.5)
	Chemotherapy	13 (14.4)	17 (11.6)
	Chemotherapy + Immunotherapy	15 (16.7)	29 (19.7)
	Targeted therapy	36 (40.0)	43 (29.3)
Alanine aminotransferase (U/L) (mean (SD))		26.36 (24.53)	27.76 (39.36)
Lung cancer diameter (cm) (mean (SD))		4.24 (2.56)	4.07 (2.63)
Total volume of pleural effusion (cm) (mean (SD))		9.94 (5.90)	8.95 (4.65)

Table S3. Essential radiomic features and formula composition.

Intercept/Feature Name	Regression coefficient
Intercept=0.795838	*β*
Inverse Variance	-0.447862
Large Dependence High Gray Level Emphasis	0.000014
Low Gray Level Emphasis	0.034752
Long Run High Gray Level Emphasis	0.000087
Skewness	0.075103
Median	0.00649
Dependence Variance	-0.05396
Small Dependence Low Gray Level Emphasis	-0.240323

β, Regression coefficient.

SUPPLEMENTARY METHODS

Methods S1: Hyperparameter tuning strategy, related to Method Details.

Logistic Regression (Baseline model)

Tuning method: Standard 5-fold cross-validation for performance evaluation (no hyperparameter tuning required as it has no tunable hyperparameters)

Evaluation metric: ROC AUC

1. Decision Tree

Hyperparameter ranges:

tree_depth: 3 – 7

min_n: 5 – 10

cost_complexity: 10^{-6} – 10^{-1}

Tuning method: 5-fold cross-validation with random grid search (grid_random), sampling 5 parameter combinations.

Evaluation metric: ROC AUC (primary), Accuracy, PR AUC.

Selection criterion: The parameter combination with the highest ROC AUC and lowest complexity was selected using select_by_one_std_err.

2. Random Forest

Hyperparameter ranges:

mtry: 2 – 10

trees: 200 – 500

min_n: 20 – 50

Tuning method: 5-fold cross-validation with regular grid search (grid_regular), 2 candidate values for each parameter.

Evaluation metric: ROC AUC.

Selection criterion: The parameter combination with the highest ROC AUC was selected directly.

3. XGBoost

Hyperparameter ranges:

mtry: 2 – 8

min_n: 5 – 20

learn_rate: 10^{-3} – 10^{-1}

tree_depth: 1 – 3

loss_reduction: 10^{-3} – 1

sample_size: 0.8 – 1

Tuning method: 5-fold cross-validation with random grid search (grid_random), sampling 5 parameter combinations.

Evaluation metric: ROC AUC.

Selection criterion: The parameter combination with the highest ROC AUC was selected directly.

4. Elastic Net

Hyperparameter ranges:

mixture: 0 (pure ridge) – 1 (pure LASSO)

penalty: 10^{-5} – 1

Tuning method: 5-fold cross-validation with regular grid search (grid_regular), 5 candidate values for mixture and 10 for penalty.

Evaluation metric: ROC AUC.
Selection criterion: Balanced model complexity and performance using select_by_one_std_err.
5. Radial Support Vector Machine (rSVM)
Hyperparameter ranges:
cost: 10^{-5} – 10^{5}
rbf_sigma: 10^{-4} – 10^{-1}
Tuning method: 5-fold cross-validation with regular grid search (grid_regular), 2 candidate values for cost and 3 for rbf_sigma.
Evaluation metric: ROC AUC.
Selection criterion: The parameter combination with the highest ROC AUC was selected directly.
6. Multilayer Perceptron (MLP)
Hyperparameter ranges:
hidden_units: 15 – 24
penalty: 10^{-3} – 1
epochs: 50 – 150
Tuning method: 5-fold cross-validation with regular grid search (grid_regular), 2 candidate values for each parameter.
Evaluation metric: ROC AUC.
Selection criterion: The parameter combination with the highest ROC AUC was selected directly.
7. K-Nearest Neighbors (KNN)
Hyperparameter ranges:
neighbors: 3 – 11
Tuning method: 5-fold cross-validation with regular grid search (grid_regular), 5 candidate values.
Evaluation metric: ROC AUC.
Selection criterion: The parameter combination with the highest ROC AUC was selected directly.
General Workflow:
1.Data Preprocessing: All models shared the same preprocessing pipeline (removing ID columns, handling missing values, and encoding categorical variables).
2.Cross-Validation: 5-fold cross-validation was used to ensure robust evaluation of generalization performance.
3.Parameter Selection: ROC AUC was prioritized as the primary metric, with some models incorporating standard deviation (select_by_one_std_err) to avoid overfitting.
4.Reproducibility: A fixed random seed (set.seed(42)) was used throughout the process.
