# Supplementary material for: Development and multicenter validation of a predictive model for malignant pleural effusion recurrence
Source: iScience. 2026 Feb 17;29(3):115040. doi: 10.1016/j.isci.2026.115040 (PMC12989844; doi:10.1016/j.isci.2026.115040)
Supplement: Data S1. Ethical approval documents [file mmc3.pdf]

# 川北医学院附属医院医学伦理委员会伦理审查批件

# IRB Review Approval Notice

批件号 File Number：2022ER234-1

| | | | | |
|---|---|---|---|---|
| 项目名称<br>Study Title | 川东北地区肺结节良恶性数学预测模型的建立与验证。<br>Establishment and verification of mathematical prediction model for benign and malignant pulmonary nodules in Northeast Sichuan. | | | |
| 项目来源<br>Project source | 研究生毕业课题 | | | |
| 审查类别<br>Review type | 初始审查 Conference Review☑； 修订后审查 Revised Review□；<br>跟踪审查 Follow-up review□ | | | |
| 审查方式<br>Review way | 会议审查 Conference Review□； 快速审查 Quick Review☑ | | | |
| 主要研究者<br>Principal investigator | 蒋莉 黄语嫣 | 职 称<br>Professional title | 主任医师 | |
| 承担科室<br>Research department | 呼吸与急危重症科 | 联系电话<br>Phone No. | 13388139657 | |
| 审查日期<br>Review date | 2022-07-18 | 审查地点<br>Address | - | |
| 审核内容<br>Reviewed items | 研究方案版本号<br>Study protocol version number | V1.0 | 研究方案版本日期<br>Study protocol version date | 2022年4月7日 |
| | 知情同意书版本号<br>Informed Consent Form version number | - | 知情同意书版本日期<br>Date of informed consent form | - |
| | 其他文件<br>Other specify | 主要研究者资质 知情同意书豁免申请 | | |
| 主审委员<br>Presiding judge | 胡春梅□ 崔 曙□ 张全波□ 谢建平□ 梁 斌□ 马代远□ 任亦星□ 陈天武☑ 侯令密□ 刘凤君☑ 曾玉华□ 赵 婧 □ 杨 明□ 郭 斌□ 魏雪梅☑ 刘 辉□ 周士逵□ | | | |
| 审查结果<br>Decision: | 同意(Approved)☑； 作必要修改后同意(Conditional approved)□；<br>作必要修改后再审(Reviewde after revising)□； 不同意(Disapproved)□；<br>终止或暂停先前批准的实验(Temination/Suspension)□ | | | |
| 年度/定期跟踪审查频率<br>Regular tracking review frequency | 该研究进行过程中将接受伦理委员会的年度/定期跟踪审查频率<br>3个月（3months)□； 6个月(6months)□； 12个月(1year)□； 不适用(NA)☑ | | | |
| 主任委员（副主任委员）<br>签字/盖章<br>Commissioner signature | [signature] | | 伦理委员会盖章<br>IRB signature | |
| 签发日期<br>Date | 2022 年 7 月 18 日 | | | |



地址：四川省南充市顺庆区茂源南路 1 号 邮编：637000 电话：0817-2262124

扫描全能王 创建
